# Communicating with society about the science behind meat and livestock production

**DOI:** 10.1093/af/vfae039

**Published:** 2025-04-05

**Authors:** Alexa J Lamm

**Affiliations:** Department of Agricultural Leadership, Education and Communication, University of Georgia, Athens, Georgia, USA

**Keywords:** audience, channel, communication, framing, source credibility

**Implications**

• Communicating with society about the science behind meat and livestock production is difficult given the lack of scientific information available online when compared with the amount of misinformation available.

• Identifying demographic and sociographic characteristics of the audience being targeted with scientific information is imperative to developing a science communication strategy.

• Framing a message to align with identified audience needs by using language acceptable to the targeted group will improve message salience.

• Sharing a properly framed message on a channel used most frequently by the target audience is imperative to the message reaching those for whom it is intended.

• Using a source, the intended audience already trusts to share a properly framed message on a channel the target audience uses will most effectively communicate the science behind meat and livestock production.

## Introduction

Innate curiosity associated with how science works should be nurtured (West and Bergstrom, 2021). How to consider evidence and determine source credibility when making decisions should be taught. Understanding how popular media channels and social media influencers can and will distort scientific conclusions to push an agenda must be recognized. Scientists must prioritize public understanding of and engagement in science. Without an informed public, policies negatively impacting how food can be grown, harvested, and delivered will be made based on misinformation delivered by untrustworthy, yet charismatic, sources. However, communicating agricultural and environmental science effectively is a difficult task given the myriads of online mainstream and social media channels used (Dobbins et al., 2021), exorbitant amounts of misinformation readily available (West and Bergstrom, 2021), and algorithms dictating the information prioritized in communication feeds.

Communicating the science behind meat and livestock production is especially challenging. While individuals are increasingly empowered by information obtained online, misinformation about livestock production practices has led to an increase in consumer concern and policy initiatives that do not consider evidence and source credibility about the information being shared. An inability to obtain empirically based scientific information supporting the requirements associated with recent farm animal confinement legislation being passed in the United States is just one example. Unfortunately, the scientific community has not been proactively communicating the science behind food production for a myriad of reasons; including a fear of criticism (Lamm et al., 2023). Most often the only scientific findings available online are in hard to access scientific journals, written at an advanced level few can interpret, and overuse technical jargon. The result is a lack of digestible scientific information available online which can be used by a layperson to inform science-based decision making and challenge misinformed policy. The scientific community must prioritize effectively sharing scientific information. Based on aggregated findings from multiple research projects, a model has been developed to assist in communicating science to reach targeted audiences.

## Agricultural and Environmental Science Communication Model

**Figure 1.**
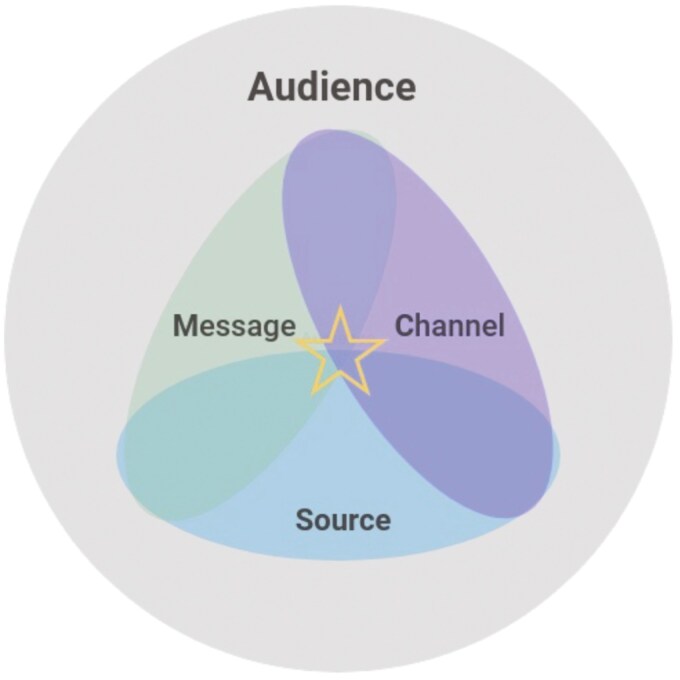
Agricultural and Environmental Science Communication (AESciComm) Model

The Agricultural and Environmental Science Communication (AESciComm) Model (Figure 1) conceptualizes how 4 concepts which interact and intersect are essential to effectively communicating science with societal actors at all levels. The model showcases how specific audiences need different messages displayed through specific channels from the right source.

### Audience

The AESciComm model is grounded in audience segmentation (Grunig, 1989) which clusters socially motivated groups within larger, diverse populations. It is commonly used to distinguish homogeneous groups of individuals who can be targeted in the same way because they have similar needs and preferences. Identifying the target audience for a science communication message and then getting to know how they communicate, where they communicate and with whom they communicate is essential to effective AESciComm. Specific audience segments can be identified by obtaining and analyzing demographic (e.g., age and gender) and sociographic (e.g., social norms such as attitudes, opinions, and interests) tendencies of specific segments of society. The effectiveness of the rest of the model is dependent upon a detailed understanding of the intended AESciComm message recipients.

### Message development

A science-based message must be developed which will resonate with the audience identified. The reading level, framing (e.g., economic, social, and environmental emphasis), and tone (e.g., level of familiarity/formality) are key things to consider when developing a scientific message. Scientists are often too formal, with their message coming off as cold or pretentious. Common mistakes include the overuse of jargon (including acronyms), overcomplicating concepts that can be broken down, using a higher reading level than the general layperson, and leaning heavily on statistics rather than using visual cues. Most people spend less than 30 s on an individual piece of information available online, including video. Therefore, a pretentious, overly complex, statistically driven message will not garner attention and be dismissed by most members of society. A science-based message will also need to align with the specific requirements of the channel being used (e.g., Instagram requires an image be uploaded to share information).

### Channel selection

A science-based message must be delivered through the channel the target audience uses to discuss and learn about the topic being shared (Dobbins et al., 2021). The scientific community is highly reliant on static websites. Members of Gen Z, individuals born between 1997 and 2012, rely on pushed media and find static websites passive and antiquated (Jones, 2023). Social media is a primary news source in the developed world with age acting as the largest differentiator on social media channel selection (Fortner et al., 2022). Facebook and YouTube dominate the space in terms of numbers of registered users but are largely utilized by older members of society (Jones, 2023). Instagram, SnapChat, TikTok, and WeChat have less registered users but are used extensively, and with more frequency, by members of the Millennial Generation (individuals born between 1981 and 1996) and Gen Z (Jones, 2023). Podcasts have also gained popularity amongst individuals from Gen Z and may serve as a channel for information delivery to this younger audience which will soon dominate the food purchasing market as they continue to enter the workforce.

When selecting the appropriate channel to use, it is important to remember it is difficult to maintain a presence on more than one or two channels at a time and repeating content across platforms will result in lower levels of engagement (Fortner et al., 2022). In addition, picking up a following on social media is difficult and takes posting consistent messages over time. Partnering with an influencer that is already successful on the channel selected, is supportive of AESciComm, and whose messages resonates with the intended audience can validate you as a source of credible information and improve the effectiveness of a communication strategy. Social media, website, and podcast analytics should be used to measure the effectiveness of channel selection and messaging attempts (Byrd et al., 2023). Reach, engagement, conversion, and sentiment are all analytics provided by most sites and can be easily interpreted to identify if messaging strategies on a specific channel are working or if adjustments need to be made to the approach.

### Source

Delivering the right message on the right channel will only work if the message is delivered by a source the target audience trusts (Fortner et al., 2022). When sharing information about livestock and meat production people do not picture a scientist or a representative of a university (Lamm et al., 2016). Rather, research has indicated the average person trusts a farmer as a source of information most when it comes to sharing information about agricultural production practices and the food they eat (Lamm et al., 2016). In addition, trust in science has been deteriorating over time (Lupia et al., 2024), so a scientist is not always a trusted source within many segments of society. People pay the most attention to those who look, think, and talk like themselves. Social norms play a major role in trust, and people must trust the deliverer of a message for it to resonate and result in some form of action. Knowing who the targeted audience will relate to and using them to deliver an AESciComm message (or at a minimum share an AESciComm message already delivered on their networks) will improve effectiveness exponentially.

## Conclusions

Society needs science to make informed decisions about livestock and meat production practices. In the absence of trusted scientific sources, people will use what they can find which is often misinformation. The scientific community can use the AESciComm Model to effectively garner the attention of specific segments of society, whether it be a consumer selecting what to feed their family at the grocery store or a decision maker impacting policy. The main points include:

- Identify demographic and sociographic characteristics of a target audience first. Effective AESciComm strategies should be built upon what interests an audience, where they go for information, and the sources they trust.
- Develop tailored, clear, concise messages which avoid jargon and align with the belief systems of the target audience.
- Use the right channel to deliver scientific information proactively rather than being passive or defensive when science is misrepresented, or policy does not align with scientific findings.
- Partner with an influencer that has already established source credibility and trust with the target audience. Have them deliver a well-crafted message aligning with their community which portrays scientific information accurately.

Research has shown most segments of society want to make informed decisions. Societal shifts and changes will require members of the scientific community remain nimble and adjust to new communication messaging strategies, channels, and methods over time. However, if scientists can meet audiences where they are and share information about scientific topics the audience cares about in a way they can understand, the innate curiosity we all associate with how science works can be nurtured and used across society to inform science-based decisions.

## About the author

**Alexa Lamm** is a professor of Science Communication in the Department of Agricultural Leadership, Communication and Education at the University of Georgia. She graduated with a degree in Animal Science (Colorado State University, 1999) and obtained a PhD in Agricultural Education and Communication from the University of Florida (2011). She is known for facilitating 2-way discussions between scientists, stakeholders, and end users of agricultural scientific advancement with the intent of using science to inform decision-making and policy formation. She received the 2020 Borlaug CAST Communication Award and is a Fellow of both the American Association for Agricultural Education and the Association for International Agricultural and Extension Education. **Corresponding author**: alamm@uga.edu
